# The Importance of Trichoscopy in Clinical Practice

**DOI:** 10.1155/2013/986970

**Published:** 2013-09-19

**Authors:** Ana Filipa Pedrosa, Paulo Morais, Carmen Lisboa, Filomena Azevedo

**Affiliations:** ^1^Department of Dermatology and Venereology, Centro Hospitalar São João EPE Porto, Alameda Prof. Hernani Monteiro, 4200-319 Porto, Portugal; ^2^Faculty of Medicine, University of Porto, Alameda Prof. Hernani Monteiro, 4200-319 Porto, Portugal

## Abstract

Trichoscopy corresponds to scalp and hair dermoscopy and has been increasingly used as an aid in the diagnosis, follow-up, and prognosis of hair disorders. Herein, we report selected cases harbouring scalp or hair diseases, in whom trichoscopy proved to be a valuable tool in their management. A review of the recent literature on this hot topic was performed comparing the described patterns with our findings in clinically common conditions, as well as in rare hair shaft abnormalities, where trichoscopy may display pathognomonic features. In our view, trichoscopy represents a valuable link between clinical and histological diagnosis. We detailed some trichoscopic patterns, complemented with our original photographs and our insights into nondescribed patterns.

## 1. Introduction

Trichoscopy (scalp and hair dermoscopy) represents a valuable noninvasive and low-cost technique, still underutilized, to rapidly differentiate clinically frequent hair disorders [1]. In their revision, Miteva and Tosti give a comprehensive and a thorough description of the usefulness of this technique in the diagnosis and follow-up of most common hair and scalp disorders, based on updated data from the literature and their personal experience [1]. We, herein, describe its application in randomly selected patients harbouring miscellaneous hair disorders, not only as a diagnostic tool, but also in monitoring treatment response or giving an insight of prognosis. 

Trichoscopy may help to distinguish scarring versus nonscarring alopecia, early androgenetic alopecia (AGA) versus telogen effluvium, and it also supports the diagnosis and predicts the prognosis of alopecia areata (AA) [1].

## 2. Materials and Methods

From our clinical practice, we randomly selected patients harbouring hair or scalp complaints in whom trichoscopy assumed particular importance in clarifying the diagnosis, differential diagnosis, and prognosis and/or in monitoring the treatment response. The trichoscopic features were compared with data from the literature, when available.

## 3. Results and Discussion

**Figure 1:**
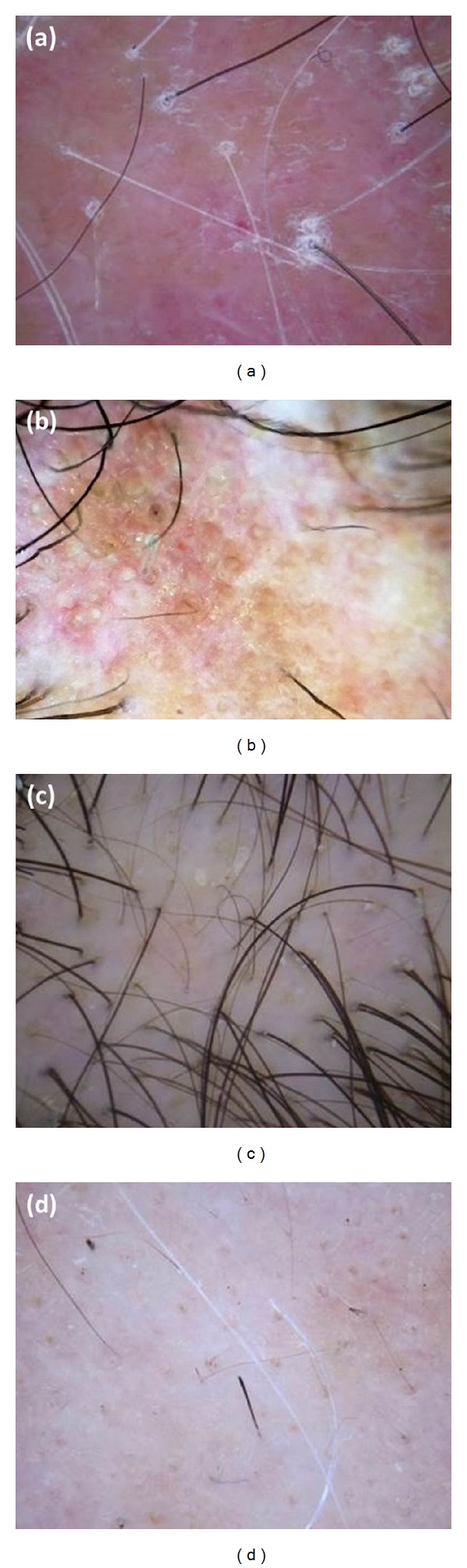
Clinically frequent hair disorders. Examples of scarring and nonscarring alopecia: (a) lichen planopilaris; (b) active scalp discoid lupus erythematosus with red follicular dots interspersed with scar areas; (c) androgenetic alopecia; (d) active alopecia areata. (a), (c), and (d) were captured with DermLite II PRO (3 Gen, San Juan Capistrano, CA, USA); (b) was captured with FotoFinder systems (Handyscope, Bad Birnbach, Germany) attached to the iPhone 4S (Apple, Cupertino, CA, USA).

Considering the conditions causing scarring alopecia, the key point seems to be the reduction or the absence of follicular orifices and the presence of peripilar casts or erythema (Figure 1(a)) [2]. Hair tufting can be seen in cases of lichen planopilaris [3]. In addition to dermoscopy, clinical presentation and physician's expertise are crucial for the diagnosis of such condition, subsequently confirmed by histopathology. There has been an effort to recognize specific features to distinguish the causes of scarring alopecia by trichoscopy. Tosti et al. [4] reported that follicular red dots seem to be a specific finding of scalp discoid lupus erythematosus (DLE), and their presence may denote disease activity, as we found in some patients with active biopsy-proven DLE (Figure 1(b)).

Regarding nonscarring alopecia, hair diameter diversity greater than 20% remains the most consistent finding in AGA in addition to perifollicular pigmentation (Figure 1(c)), whereas in telogen effluvium, there may be noticeable empty follicles and short regrowing normal thickness hairs [1]. In contrast, trichoscopy in AA may reveal uniform miniaturization of hair shafts which is most common in the remitting disease. According to other authors [1, 3], we frequently found yellow dots in both AGA and AA, although in the latter they are usually present in a greater number and may be associated with more specific markers, namely, black dots and broken and exclamation mark hairs (Figure 1(d)), which are predictors of disease activity [1, 3, 5].

Recently, we have emphasized the role of trichoscopy in the diagnosis of congenital atrichia and its distinction from other forms of childhood hair loss, especially AA universalis [6]. Trichotillomania (TTM), the compulsive desire to pull the hair, is another common cause of childhood alopecia, difficult to differentiate from AA, even with the aid of trichoscopy as both display broken hairs and black and yellow dots [5]. Some clues to TTM include the presence of scalp hemorrhages, different shaft lengths and the absence of exclamation mark hairs [7].

We had the opportunity to diagnose hair infestations with the aid of the handheld dermatoscope. Pediculosis capitis is reliably diagnosed unveiling the presence of lice nits (empty or full of nymphs) [8], and the iconographic proof of the infestation can be provided to the sceptic patients or their parents.

**Figure 2:**
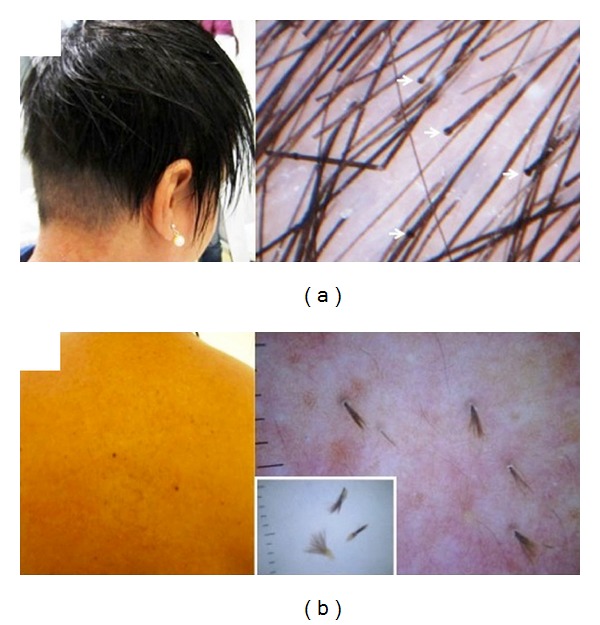
Rare hair shaft abnormalities. (a) Trichorhexis invaginata in a patient with Comèl-Netherton syndrome. Clinically hair appears sparse, brittle, and short; trichoscopy shows nodes along the hair shafts (white arrows). (b) Pili multigemini on the back. Dermoscopy highlighting the multiple hair shafts emerging from the same follicular ostia. Dermlite II Pro (3 Gen, San Juan Capistrano, CA, USA).

Genetic hair shaft abnormalities may be visualized by trichoscopy avoiding the need to pluck or cut hairs to perform light microscopy [9]. We display an example of trichorrhexis invaginata in a patient with Comèl-Netherton syndrome, highlighting the multiple nodes along the hair shafts, giving the appearance of “bamboo hair” (Figure 2(a)), which is considered pathognomonic for the disease [9].

Additionally, a case of nevoid pili multigemini in a patient complaining of skin roughness of the back was diagnosed with trichoscopy that enabled the recognition of multiple hair shafts emerging from the same follicle. After plucking the hairs, dermatoscopy suggested their true multigeminate nature coming from a single papilla and looking identical in a brush-like pattern (Figure 2(b)), different from the more commonly found compound follicles [10, 11]. 

## 4. Conclusions

In conclusion, we aimed to have briefly presented our experience in the daily clinical practice using a handheld dermatoscope coupled with a digital camera (or a smartphone) in the study of hair and scalp disorders. Trichoscopy represents a valuable link between clinical and histologic diagnoses. We have found reproducibility of many trichoscopic patterns complemented with our dies and original photographs.
